# Degradation Characteristics of Color Index Direct Blue 15 Dye Using Iron-Carbon Micro-Electrolysis Coupled with H_2_O_2_

**DOI:** 10.3390/ijerph15071523

**Published:** 2018-07-19

**Authors:** Bo Yang, Yingying Gao, Dengming Yan, Hui Xu, Junfeng Wang

**Affiliations:** Environmental Protection Engineering Center for Pollution Treatment and Control in Textile Industry, College of Environmental Science and Engineering, Donghua University, Shanghai 201620, China; yangbo@dhu.edu.cn (B.Y.); 18302152318@163.com (Y.G.); xuhui911@163.com (H.X.); junfengwang805@163.com (J.W.)

**Keywords:** dye wastewater, iron-carbon micro-electrolysis, kinetics, degradation mechanism

## Abstract

Currently, many industrial dyes are discharged into the environment in China, leading to serious water pollution. However, synthetic organic dyes in industrial effluents cannot be degraded by conventional wastewater treatment methods. Consequently, it is necessary to develop new environmentally friendly technologies to completely mineralize these non-biodegradable compounds. In this study, 300 mg/L typical Color Index (CI) Direct Blue 15 (benzidine disazo) in simulated dye wastewater was degraded by iron-carbon micro-electrolysis coupled with H_2_O_2_ to explore its decolorization, total organic carbon (TOC) removal rate, and degradation characteristics. Under the optimal degradation conditions (Fe/C = 2:1, pH = 3, 60-min reaction, 2 mL/L H_2_O_2_ (added in three aliquots), 300 mg/L dye), the TOC removal rate and the level of dye decolorization attained 40% and 98%, respectively. In addition, the degradation kinetics indicated that the iron-carbon micro-electrolysis process coupled with H_2_O_2_ followed first-order reaction kinetics. A degradation pathway for CI Direct Blue 15 was proposed based on the analysis results of treated wastewater obtained using UV-Vis spectrophotometry and gas chromatography–mass spectrometry (GC-MS). This study provides an efficient and economical system for the degradation of non-biodegradable pollutants.

## 1. Introduction

A large amount of synthetic organic dyes are applied in many fields, such as textiles, plastics, food, paper, printing, leather, cosmetics, pharmaceuticals, coatings, and others [1,2,3]. It is estimated that the annual output of dyes exceeds 100,000 tons. About 10% of the dye output is discharged into the environment. Dye effluents are characterized by high color, high organic content, complex composition, and poor biodegradability [4,5,6]. Among synthetic organic dyes, azo dyes are the most widely used, and they account for 60–70% of the total consumption of dyes. Furthermore, it was proven that most azo dyes and their metabolites can generate toxic, carcinogenic, mutagenic, and teratogenic effects on human health and the environment [7,8,9,10]. According to the Ecological and Toxicological Association of Dyes and Organic Pigments Manufacturers (ETAD) survey on about 4000 kinds of dyes, the toxicity of diazo direct dyes is the highest [11]. The intermediate aromatic amines and acrylamides generated in the metabolism process of benzamine-derived azo dyes have serious carcinogenic effects [12,13]. Therefore, azo-dye wastewater is a major concern, and the removal of azo dyes is extensively explored.

The removal methods of dyes from wastewater can be roughly classified as physicochemical, chemical, and biological methods [14,15]. However, these methods have some limitations, such as high processing costs, complex processes, high sludge production, and secondary pollution effects. It is necessary to develop new economic and effective technologies to treat dye wastewater [16,17]. Various advanced electrochemical oxidation processes are applied in China, including electro oxidation (EO, also known as electrochemical oxidation or anodic oxidation), electrocoagulation, and electro-Fenton-based (EF) methods, to treat dye wastewater. These electrochemical processes achieved good results; however, they are expensive [18,19,20,21]. Iron-carbon micro-electrolysis coupled with H_2_O_2_ is an advanced oxidation process (AOP), characterized by low processing cost, low energy consumption, long service life, simple operation, and convenient maintenance [22,23]. However, the decolorization effect and degradation mechanism of azo-dye wastewater is yet to be elucidated using this technology.

In this study, the degradation characteristics of benzidine amide azo dyes were studied using iron-carbon micro-electrolysis coupled with H_2_O_2_. The degradation of the dye and its intermediates was estimated using UV-Vis spectrophotometry and gas chromatography–mass spectrometry (GC-MS). This study provided a basis for estimating the feasibility of this technique for the treatment of printing and dye wastewaters.

## 2. Materials and Methods

### 2.1. Experimental Materials

Activated carbon (Shandong Yingke Linchuan Environmental Protection Technology Co., Ltd., Shandong, China) with a particle size of 0.5–1 mm was used in the experiment. The activated carbon was pretreated to eliminate any potential adsorption. The activated carbon was immersed in 300 mg/L Color Index (CI) Direct Blue 15 dye wastewater for 72 h, and the dye wastewater was replaced by fresh wastewater every 12 h so that the activated carbon reached adsorption saturation. Then, the treated activated carbon was dried in an oven at 105 °C.

Iron scraps with a particle size of 1–2 mm were used in the experiment. Firstly, iron scraps were soaked in 20% NaOH solution for 90 min to remove surface oil, and were rinsed with deionized water until a pH of 7 was achieved, before being put into 10% H_2_SO_4_ for 60 min to remove surface oxides.

**Figure 1:**
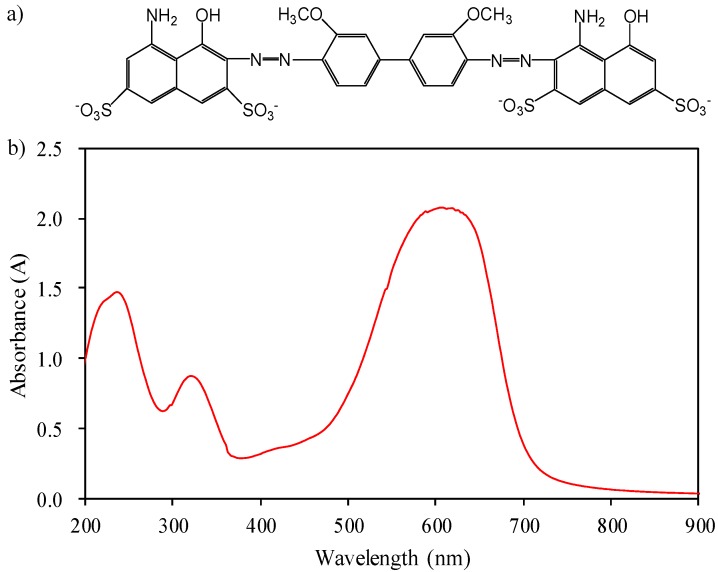
Chemical structure (**a**) and absorbance spectrum of Color Index (CI) Direct Blue 15 (**b**).

Water samples were prepared using a certain concentration of simulated CI Direct Blue 15 dye (Xiamen Haiwei Technology Co., Ltd., Xiamen, China) wastewater in the laboratory for experimentation. Dye molecules (C_34_H_24_N_5_Na_4_O_16_S_4_) contained an azo structure (Figure 1a), and had a relative molecular mass of 992.8. The dye color was blue, with a maximum absorption wavelength of 602 nm, as indicated by the UV-Vis spectrum (Figure 1b). Therefore, this wavelength was used to determine the concentration of CI Direct Blue 15 in wastewater.

### 2.2. Experimental Methods

**Figure 2:**
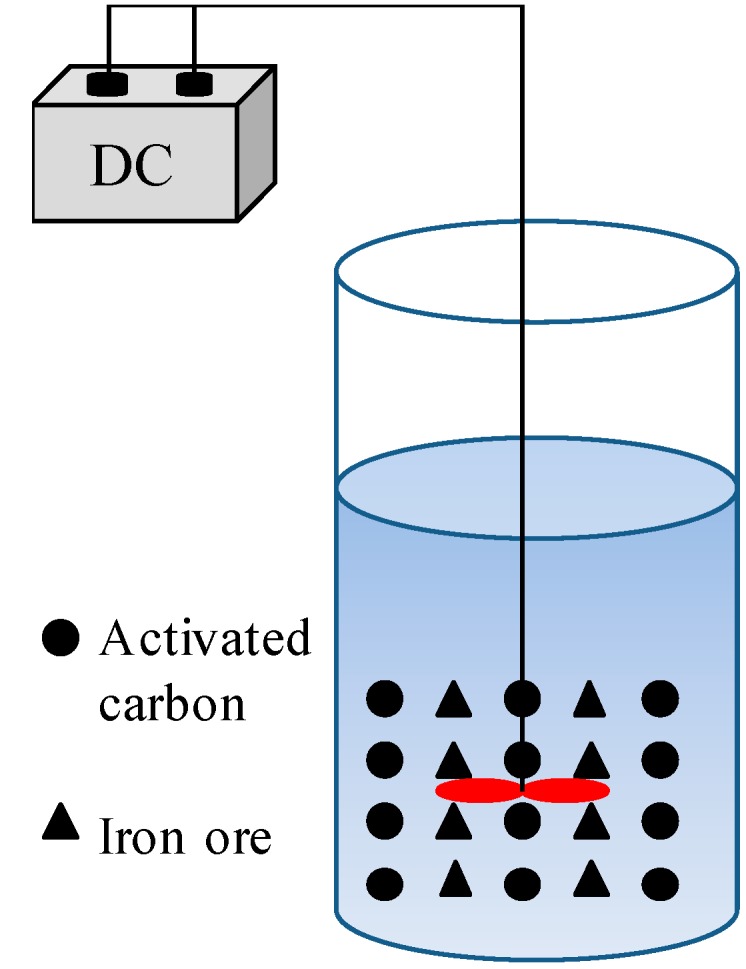
Schematic diagram of the experimental device.

The reactor was made of Perspex (without cap) with a diameter of 11 cm, a height of 14 cm, and a reaction volume of 1.3 L. The reactor was equipped with a mixing device (MY3000-6N, Wuhan Meiyu Instrument Co., Ltd., Wuhan, China.), allowing iron and active carbon to be fully mixed, thereby increasing the reaction rate. In addition, the mixing device could increase the amount of dissolved oxygen in wastewater so that Fe^2+^ produced by Fe/C electrolysis was oxidized into Fe^3+^, forming Fe(OH)_3_, which is conducive to the flocculation of organic compounds. The stirring speed was set to 150 rpm, and 800 mL of simulated CI Direct Blue 15 dye wastewater was added to the reactor. Then, 30% H_2_O_2_ solution was added. Under the conditions of pH = 3 and Fe/C = 2:1, the influences of reaction time (15, 30, 45, 60, 75, or 90 min), initial dye concentration (100, 200, 300, 400, or 500 mg/L), dosage of 30% H_2_O_2_ (0.5, 1.0, 1.5, 2.0, 2.5, or 3.0 mL/L), and 2.0 mL/L 30% H_2_O_2_ dosing methods (added in one, two, or three aliquots) on the decolorization and degradation process of CI Direct Blue 15 dye wastewater were investigated. The experimental device is shown in Figure 2.

### 2.3. Experimental Apparatus and Analysis Methods

A coagulation mixer (Meiyu Brand, MY3000-6N, Wuhan Meiyu Instrument Co., Ltd., Wuhan, China), pH meter, centrifuge, UV-Vis spectrophotometer (UV-7504PC, Shanghai Precision Instrument Co., Ltd., Shanghai, China), total organic carbon (TOC) tester (multi N/C 3100, Jena Analytical Instruments AG, Jena, Germany), rotary evaporator, and gas chromatography–mass spectrometer (Agilent GC7890B/MS5977A, Suzhou Maikesi Instrument Equipment Co., Ltd., Suzhou, China.) were used to determine the experimental parameters. The analysis parameters and methods are given in Table 1.

The TOC and dye concentrations in water samples were measured after centrifugation (6000 rpm, 10 min). 

**Figure 3:**
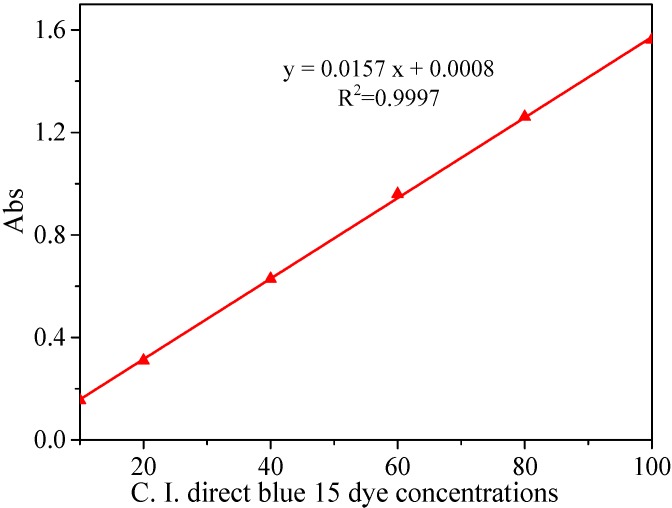
Standard curve of CI Direct Blue dye absorbance and concentration.

The rate of decolorization was calculated on the basis of the Lambert–Beer law. Various CI Direct Blue 15 dye concentrations were prepared, and their absorbance values were measured at a wavelength of 602 nm. With the CI Direct Blue 15 dye concentrations as the abscissa and the absorbance values as the ordinate, a standard curve was drawn (Figure 3). The correlation coefficient was R^2^ = 0.9997, and the linear equation was y = 0.0157x + 0.0008 (y: Absorbance; x: CI Direct Blue 15 concentration). As such, the rate of decolorization was calculated as follows:

R = (A_0_ − A_e_)/A_0_ × 100% = (C_0_ − C_e_)/C_0_ × 100%, (1)

where A_0_ and A_e_ are the absorbance values of CI Direct Blue 15 dye wastewater at time 0 and reaction time *t*, respectively. C_0_ (mg/L) and C_e_ (mg/L) are the CI Direct Blue 15 dye concentrations at time 0 and reaction time *t*, respectively.

The samples were prepared for GC-MS analysis as described below.

Firstly, 500 mL of the water samples obtained after centrifugation were extracted with CH_2_Cl_2_ six times (three times under acidic conditions and three times under alkaline conditions). Then, the samples were filtered through anhydrous Na_2_SO_4_. Finally, 150 mL of the filtered samples were extracted. The extraction was performed according to the method described by the United States (US) National Environmental Protection Agency. A separatory funnel (EPA3510C) was used for liquid–liquid extraction [24]. The extracted samples were evaporated to a volume of 5 mL via rotary evaporation, and were then stripped with nitrogen to a volume of 2 mL. The concentrated samples were used for the analysis of degradation products via GC-MS.

The GC-MS analysis of organic compounds was performed according to JY/T021-1996 Gas Chromatography Determination Method and GB/T6041-2002 General Principles of Mass Spectrometry (GC-MS). An Agilent GC7890B/MS5977A was used for analysis with a column (Agilent HP-5MSUI 30 m × 0.25 mm × 0.25 μm), under conditions of an injection volume of 1.0 μL, an inlet temperature of 280 °C, a transmission line temperature of 280 °C, an ion source temperature of 230 °C, a quadrupole temperature of 230 °C, and a column temperature of 40 °C (for 4 min) before being raised at 8 °C/min until a temperature of 300 °C was achieved, and maintained for 12 min. Furthermore, a full scan mode was used, as well as a scanning range of 30–1000 u, an EM (Electron multiplier) voltage of 1079 V, an EI (Electron ionization) MS ionization mode, a filament energy of 70 eV, and helium as the carrier gas, at a flow rate of 1.0 mL/min. The organic compounds were qualitatively determined based on the NIST2014 library, and partial compounds were confirmed based on the retention time of the standard compounds.

## 3. Results and Discussion

### 3.1. Effects of Multiple Factors on Dye Degradation

#### 3.1.1. Effects of Initial Dye Concentration on the Decolorization and TOC Removal Efficiency of Dye Wastewater

**Figure 4:**
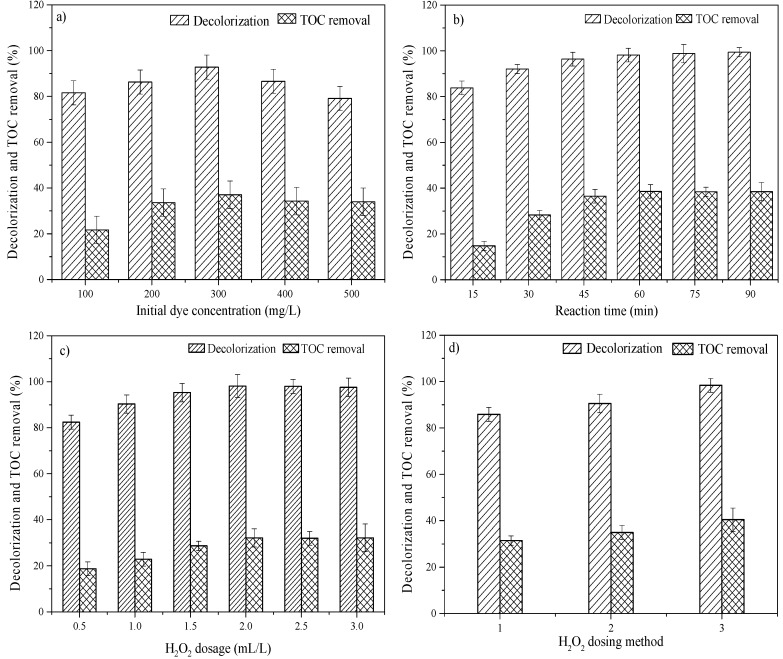
Effects of operational parameters on the degradation of CI Direct Blue 15 dye using iron-carbon micro-electrolysis coupled with H_2_O_2_: (**a**) effect of initial dye concentration (pH = 3, Fe/C = 2:1, reaction time = 60 min, H_2_O_2_ dosage = 2 mL/L, H_2_O_2_ dosing in three aliquots); (**b**) effect of reaction time (pH = 3, Fe/C = 2:1, initial dye concentration = 300 mg/L, H_2_O_2_ dosage = 2 mL/L, H_2_O_2_ dosing in three aliquots); (**c**) effect of H_2_O_2_ dosage (pH = 3, Fe/C = 2:1, initial dye concentration = 300 mg/L, reaction time = 60 min, H_2_O_2_ dosing in three aliquots); (**d**) effect of H_2_O_2_ dosing method (pH = 3, Fe/C = 2:1, initial dye concentration = 300 mg/L, reaction time = 60 min, H_2_O_2_ dosage = 2 mL/L).

The initial dye concentration has an effect on its decolorization and level of TOC removal [25]. Under conditions of pH = 3, Fe/C = 2:1, and a dosage of hydrogen peroxide of 2 mL/L, the concentration of added dye was changed. The concentrations of dye and TOC in wastewater were measured after 60 min, and the results are shown in Figure 4a. 

The decolorization and level of TOC removal increased as the dye concentration increased from 100 mg/L to 300 mg/L. After the initial dye concentration was increased above 300 mg/L, both the decolorization and TOC removal rate decreased. This experimental phenomenon is similar to that observed in the MEC(Microbial Electrolysis Cell)-Fenton and classical Fenton processes [26]. The changing trend is interpreted below.

The removal of CI Direct Blue 15 dye was based on the strong oxidation function of ·OH, which degraded the molecular structure of the dye and broke the chromophore, thus resulting in chromaticity. When the dye concentration was low, the number of produced hydroxyl radicals was larger than that of the dye molecules in the water body, and these hydroxyl radicals could break the azo bonds of the chromophore groups of most dye molecules. Therefore, the decolorization rate of the dye wastewater was higher. However, when the initial dye concentration was increased above 300 mg/L, which potentially exceeded the limit of the hydroxyl radical oxidation capacity, the decolorization efficiency of dye wastewater was reduced.

The removal of TOC also increased at first and then decreased. At a low initial concentration of influents, the amount of ·OH produced was sufficient to degrade the intermediate products of dye degradation. As such, the removal level of TOC was high. However, the higher the initial concentration of dye, the more occupied the active sites became. The number of active sites of Fe^2+^/Fe^3+^ for decomposing H_2_O_2_ was decreased. Therefore, the formation rate of H_2_O_2_ was lower, and the TOC removal rate decreased [27,28]. In addition, with an increase in initial concentration, more radical scavengers (${\mathrm{SO}}_{4}^{2-}$, ${\mathrm{CO}}_{3}^{2-}$, ${\mathrm{HCO}}_{3}^{-}$) were generated to react with ·OH, thus causing an adverse effect on the mineralization process.

In addition, the current density increased with increasing dye concentration. This was similar to electro-Fenton and photoelectro-Fenton processes, where relatively higher current density was beneficial for the degradation of Orange G [29]. However, when the concentration was too high, the number of small dye molecules in the wastewater exceeded the limit of oxidation ability, resulting in a small number of small molecules left in the wastewater after the destruction of the dye. Therefore, the TOC removal rate decreased as the concentration increased.

In summary, at an appropriate initial dye concentration, iron-carbon micro-electrolysis coupled with H_2_O_2_ addition produced the optimal treatment effect. The experimental results suggested an initial dye concentration of 300 mg/L was the optimal treatment concentration.

#### 3.1.2. Effects of Reaction Time on Decolorization and TOC Removal Efficiency 

Figure 4b shows the effects of reaction time on decolorization and TOC removal efficiency in dye wastewater. The reaction time was 15, 30, 45, 60, 75, or 90 min. The decolorization increased to 98% after 60 min. When the reaction time increased above 60 min, the rate of decolorization increased slowly. The removal of TOC was relatively obvious in the beginning of the reaction, and reached 39% after 60 min. When the reaction time increased above 60 min, the removal of TOC did not increase. It shows that the degradation of the dye was due primarily to the cleavage of its chromophoric group. When the decolorization reaction was substantially completed, the mineralization of the intermediate product began. This inference could also be observed from the experimental phenomenon; as the reaction time progressed, the solution faded from dark blue. The changing trend is interpreted below.

During the decolorization process by electrolysis, the dye molecules were involved in the electrode reaction and their structures were changed. The chromogenic group, –N=N–, obtained electrons from the electrode. The azo bonds broke, and the conjugated system of original dye molecules was damaged [30]. The electrochemical reaction between dye molecules and the iron electrode caused the decolorization of the solution, and the Fe^2+^ produced during the ferric-carbon electrolysis process reacted with the hydrogen peroxide to generate hydroxyl radicals with strong oxidization capability. In this way, the dye macromolecules were broken to produce small molecules, and the luminescent groups were degraded and decolorized. Therefore, the decolorization effect of the dye was more obvious. However, during the decolorization reaction, the iron consumption gradually increased and the hydrogen peroxide was consumed completely. Finally, a large number of Fe^2+^ ions were dissolved and oxidized into Fe^3+^. Therefore, the decolorization rate decreased, and even the phenomenon of color recovery occurred. During the decolorization process, the decolorization firstly increased and then remained stable.

The removal of TOC increased significantly at the beginning of the reaction, before increasing slowly. The phenomenon might be interpreted according to the theory of iron-carbon microelectrolysis, where a large amount of Fe^2+^ is produced in the initial stage of the reaction, and Fe^2+^ reacts with the added H_2_O_2_ to increase the number of hydroxyl radicals generated, which promotes the degradation of the dye. After 60 min of reaction, ·OH was consumed completely, and the mineralization rate of the dye decreased. In addition, the dyes consisting of macromolecules could not be oxidized completely into CO_2_ and H_2_O, and potentially produced intermediates such as aromatic rings. During the catalysis process of ·OH, aromatic rings are more easily broken than azo bonds [31,32]. Therefore, the decolorization process was more significant than the mineralization process.

The data on decolorization and TOC removal indicated that the optimal treatment time was 60 min. Bouzayani et al. [33] obtained similar results through the heterogeneous Fenton degradation of polyvinylamine sulfonate anthrapyridone dyes.

#### 3.1.3. Effects of H_2_O_2_ Dosage on the Decolorization and TOC Removal Efficiency

Most previous studies indicated that the addition of H_2_O_2_ had an impact on the degradation of the dye. The doses of H_2_O_2_ adopted in this experiment were 0.5, 1.0, 2.0, 2.5, and 3.0 mL/L. The effects of H_2_O_2_ dosage on dye degradation are shown in Figure 4c.

Both the decolorization and TOC removal increased with an increase in the dosage of H_2_O_2_, and peaked at a dosage of 2.0 mL/L H_2_O_2_. When the dosage of H_2_O_2_ increased above 2.0 mL/L, TOC removal remained unchanged. Similar results were reported by many researchers [34,35,36]. 

Considering the available literature, it was hypothesized that, when the concentration of H_2_O_2_ was low, the number of hydroxyl radicals could not oxidize the dye molecules completely, and when the H_2_O_2_ concentration was high, more hydroxyl radicals were formed, thus significantly improving the decolorization and TOC removal. However, excessive hydrogen peroxide could not produce more free radicals via decomposition, and Fe^2+^ was oxidized rapidly into Fe^3+^ at the beginning of the reaction, thus consuming partial catalysts for the oxidation reaction of Fe^3+^. Therefore, both the consumption of H_2_O_2_ and the inhibited generation of hydroxyl radicals slowed the decolorization and TOC removal. In addition, according to the Fenton reaction principle (Fe^2+^ + H_2_O_2_ = Fe^2+^ + ·OH + OH^−^, H_2_O_2_ + ·OH = HO_2_· + H_2_O), excessive H_2_O_2_ will react with ·OH to produce ·OOH radicals [37]. The oxidation capacity of ·OOH is much lower than that of ·OH radicals, thus leading to an invalid decomposition of H_2_O_2_, decreasing the decomposition of the dyes [38]. An appropriate amount of hydrogen peroxide could increase the utilization of oxidants and promote the decolorization and TOC removal of dye wastewater. 

#### 3.1.4. Effects of H_2_O_2_ Addition Method on Decolorization and TOC Removal

The addition method of hydrogen peroxide also affected the performance of the iron-carbon micro-electrolysis coupled with H_2_O_2_. Most studies reported that a repeated addition of hydrogen peroxide could improve the removal efficiency of organic matters [39].

Figure 4d shows the decolorization and TOC removal for H_2_O_2_ addition performed in one, two, or three aliquots. The decolorization and TOC removal slightly increased when the number of H_2_O_2_ additions was increased from one to three. However, after excluding the standard deviation of the data, the increase was not significant.

When H_2_O_2_ was added in batches, the invalid decomposition of H_2_O_2_ was reduced, and the ratio of [H_2_O_2_]/[Fe^2+^] was always relatively low. The catalyst concentration was relatively high, and the ·OH yield was high, thus improving the utilization rate of H_2_O_2_. Moreover, the overall oxidation effect, decolorization, and TOC removal rate were also significantly improved. Therefore, H_2_O_2_ can be added in batches in dye wastewater treatment. 

### 3.2. Analysis of the Reaction Kinetics

**Figure 5:**
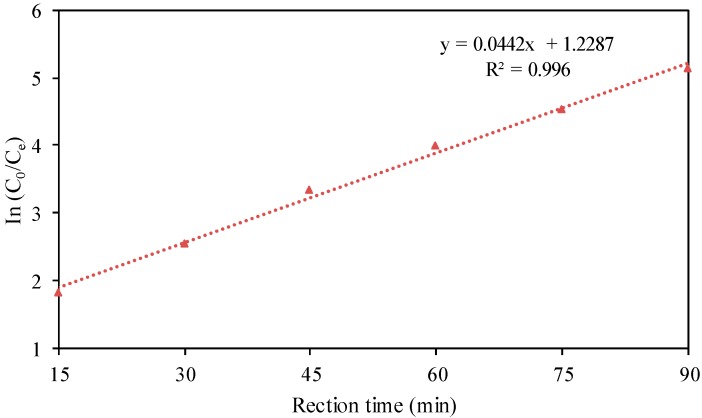
Kinetics analysis of reaction.

The kinetic characteristics of the reaction with changes in the concentration of dye wastewater and in the reaction time are shown in Figure 5. 

As shown in Figure 5, a straight line of ln(C_0_/C_e_) versus reaction time was achieved, with a correlation coefficient of R^2^ = 0.996. Therefore, the use of iron-carbon micro-electrolysis coupled with H_2_O_2_ for the treatment CI Direct Blue 15 dye wastewater was consistent with first-order kinetics.

### 3.3. Analysis of the Degradation Mechanism of CI Direct Blue 15 Dye 

**Figure 6:**
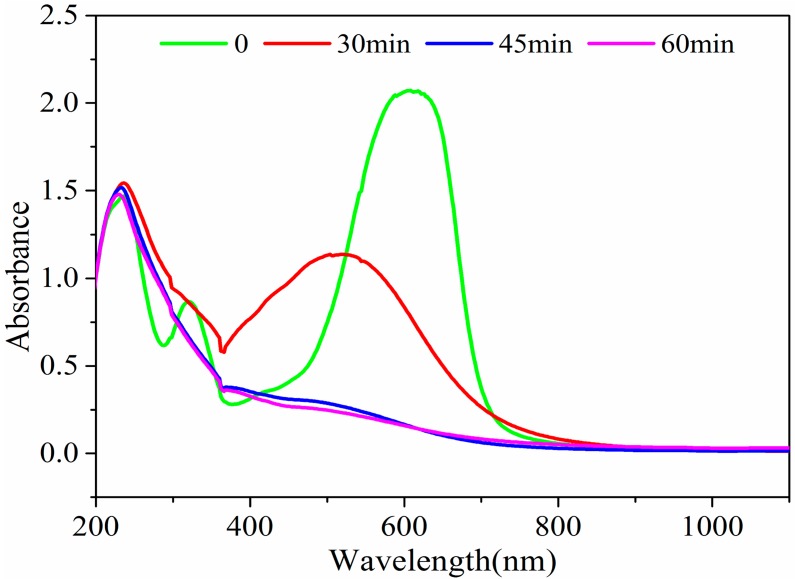
Full-band spectrum of the dye degradation process.

The maximum absorption wavelength of CI Direct Blue 15 dye is 602 nm. The full-band spectrum between 200 nm and 1100 nm (Figure 6) was recorded after 30 min, 45 min, and 60 min during the degradation process to analyze the treatment performance.

During the degradation reaction, the absorbance of the peak in the visible region gradually decreased (Figure 6), indicating that the chemical bond was broken. After 60 min of reaction, the original peak disappeared, indicating that the chemical bond was completely broken. The result was consistent with the decolorization trend of the solution. The consistency indicated that the broken chemical bond was the bond of chromophore group, the azo bond (–N=N–). In the ultraviolet region, the peak change was small and a new peak appeared, indicating that the intermediate products were produced after the chromophore groups of dye macromolecules were broken. The intermediate products could be further degraded. The above analysis indicates that iron-carbon micro-electrolysis coupled with H_2_O_2_ addition can effectively treat CI Direct Blue 15 dye wastewater. 

**Figure 7:**
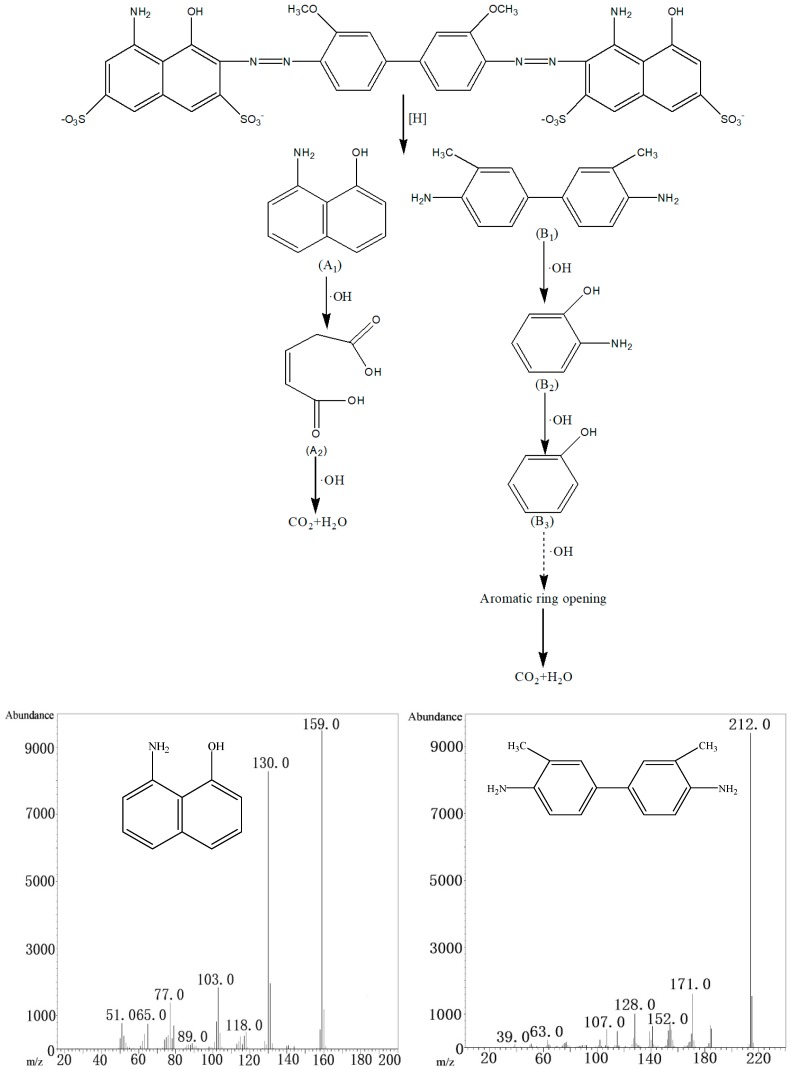

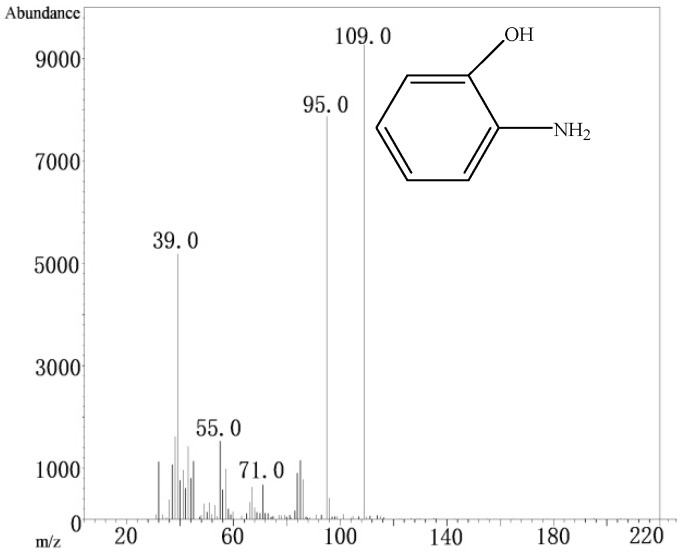
Possible degradation pathways of CI Direct Blue 15 dye

In order to further investigate the degradation process of the intermediates of the dye, the resulting supernatant was analyzed using GC-MS (Table 2). There were five main degradation products of CI Direct Blue 15. From these products, a possible degradation pathway of CI Direct Blue 15 dye could be deduced (Figure 7). The degradation of the dye was mainly caused by the [H] and ·OH produced by the iron-carbon micro-electrolysis coupled with H_2_O_2_ addition [40]. 

As shown in Figure 7, the azo bond (–N=N–) was first attacked by the [H] produced from the internal electrolysis, and the –N=N– bond underwent a fracture hydrogenation reaction. The CI Direct Blue 15 decomposed into A_1_ (1-amino-8-naphthol) and B_1_ (3,3′-dimethylbenzidine). The hydroxyl radical (·OH) further oxidized the A_1_ product (1-amino-8-naphthol), and the benzene ring opened to form A_2_ (anti-pentenoic acid), which was presumed to produce CO_2_ and H_2_O under the oxidation action of ·OH. B_1_ (3,3′-dimethylbenzidine) was also attacked by hydroxyl radicals, and was then oxidized into B_2_ (2-aminophenol), which produced B_3_ (phenol) under the oxidation of ·OH. Phenol was speculated to degrade into CO_2_ and H_2_O through a series of oxidation reactions. 

## 4. Conclusions

Under optimal conditions for the iron-carbon micro-electrolysis coupled with H_2_O_2_ addition, the TOC removal rate and the decolorization of CI Direct Blue 15 dye reached levels of 40% and 98%, respectively, after 60 min of treatment. The degradation pathway of CI Direct Blue 15 dye seems to include the main intermediates of 1-amino-8-naphthol and 3,3′-dimethylbenzidine. These compounds were completely broken into anti-pentenoic acid and phenol, which are speculated to degrade into CO_2_ and H_2_O through a series of oxidation reactions. Thus, treatment with iron-carbon micro-electrolysis coupled with H_2_O_2_ addition is a promising method for the removal of textile dye wastewater. Furthermore, this study provides the favorable conditions for subsequent biological treatment. 

## Figures and Tables

**Table 1:** Analysis parameters and methods.

Parameters	Analysis Methods
Total organic carbon (TOC)	Combustion oxidation—non-dispersive infrared absorption method
Dye concentration	UV-Vis spectrophotometry
Intermediate products	Gas chromatography–mass spectrometry (GC-MS)

**Table 2:** GC-MS results under optimum conditions.

Intermediate Products	Chemical Structures	Retention Time (min)	*m*/*z*	Similarity of Intermediate Products
1-amino-8-naphthol	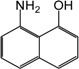	12.139	159.0	97%
3, 3′-dimethylbenzidine	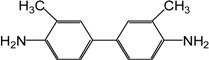	25.123	212.0	98%
anti-pentenoic acid	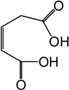	25.494	130	91%
2-aminophenol	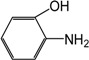	26.291	109.0	96%
phenol	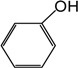	27.398	94	98%
